# Flow enhancement of water-based nanoparticle dispersion through microscale sedimentary rocks

**DOI:** 10.1038/srep08702

**Published:** 2015-03-03

**Authors:** Haiyang Yu, Youwei He, Peng Li, Shuang Li, Tiantian Zhang, Elena Rodriguez-Pin, Song Du, Chenglong Wang, Shiqing Cheng, Christopher W. Bielawski, Steven L. Bryant, Chun Huh

**Affiliations:** 1 MOE Key Laboratory of Petroleum Engineering, China University of Petroleum Beijing, Beijing. 102249, P.R. China; 2 Department of Petroleum and Geosystems Engineering, University of Texas at Austin, Austin, TX. 78712, USA; 3 Department of Petroleum Engineering, Texas A & M University, Collage Station, TX. 77843, USA; 4 Yanchang Oilfield Co., Ltd. Yan'an, Shanxi. 716000, P.R. China; 5 Department of Chemistry and Biochemistry, University of Texas at Austin, Austin, TX. 78712, USA

## Abstract

Understanding and controlling fluids flow at the microscale is a matter of growing scientific and technological interest. Flow enhancements of water-based nanoparticle dispersions through microscale porous media are investigated through twelve hydrophilic sedimentary rocks with pore-throat radius between 1.2 and 10 *μ*m, which are quantitatively explained with a simple model with slip length correction for Darcy flow. Both as wetting phase, water exhibited no-slip Darcy flow in all cores; however, flow enhancement of nanoparticle dispersions can be up to 5.7 times larger than that of water, and it increases with the decreasing of pore-throat radius. The experimental data reveals characteristic slip lengths are of order 500 and 1000 nm for 3M® and HNPs-1 nanoparticles, respectively, independent of the lithology or nanoparticle concentration or shear rate. Meanwhile, the phenomenon of flow degradation is observed for HNPs-2 nanoparticles. These results explore the feasible application of using nanoparticle dispersions to control flow at the microscale.

Usual no-slip boundary condition, that is, zero fluid velocity at the motionless surface, is not universal. That liquid molecules can slip, resulting in a non-zero velocity at solid surface, has been proved by experiments and simulations^1^,^2^,^3^. Understanding fluids flow at different scale is a matter of growing scientific and technological interest. Many researches were conducted to investigate slip flow of fluids at nanoscale, especially after remarkable flow enhancements (>10^3^) of water in carbon nanotubes were reported^4^,^5^. This unexpected flow enhancement explores the feasibility of potential applications of slip flow in biology^6^,^7^,^8^, tribology^9^, high resolution printing^10^, and high efficiency seawater desalination^11^,^12^. Several studies have indicated that the slip flow at nanoscale strongly depends on the morphology, chemistry^13^,^14^, and hydrophilicity (contact angle)^15^,^16^,^17^,^18^ of the stationary solid surface. The effect of flow channel radius on flow enhancement was also investigated. Rogers *et al*.^15^ conducted experiments in silanized flat surface with a water contact angle of 83 degrees, and found no-slip flow at boundary for toluene and slip flow for water with a constant slip length of 63 ± 3 nm, meanwhile, slip flow was enhanced in hydraulic channel with smaller radius and flow enhancement is negligible when the channel radius is in micron. Fluid flow at nanoscale does not always result in flow enhancement. Apparent viscosity of nanoconfined water can be orders of magnitudes larger than that of bulk water at hydrophilic surfaces^16^,^19^,^20^,^21^,^22^, and it greatly decreases when surfaces are increasingly hydrophobic^16^, indicating hydrophobic surface is benefit for water flow enhancement at nanoscale. Other experiments of slip flow were performed in microscale channels or capillary tubes; however the results are in remarkable disagreement. Slip lengths were about 30 nm for two different flow studies in micro-scale channels^23^,^24^ and capillary tube^25^. In microchannels, slip lengths were 50 nm^26^ in one case and 1 *μ*m^27^ in another case.

Nanomaterials show many special physical properties with small sizes and large surface areas, and nanotechnology attracts more and more interests in the past decade. Nanomaterials also have potential application in upstream oil industry, i.e., paramagnetic nanoparticles for formation evaluation and oil saturation determination in large volumes of oil reservoirs by the detection of the water-oil menisci in reservoir rocks and utilizing the concept of enhancing magnetic resonance imaging^28^,^29^,^30^. Besides, the nanoparticle stabilized emulsions and foams can be used as conformance control agents for enhanced oil recovery^31^,^32^, CO_2_ flooding^33^, and CO_2_ sequestration^34^. A prerequisite of applying nanomaterials successfully in oil reservoirs is to understand the transportability and fluid flow behaviors of nanomaterials in rocks. During our research on transport and retention of nanoparticle in reservoir rocks, the phenomenon of flow enhancement at micro-scale was unintentionally discovered. Series of nanoparticle transport experiments were thus conducted and systematically investigated the flow enhancement of nanoparticle dispersion through reservoir rocks.

In this work, we systematically evaluated the flow enhancement during nanoparticle dispersion transport through reservoir rocks by conducting core flood experiments. Water-based nanoparticle dispersions, including one hydrophilic nanoparticle sample and two hydrophobic nanoparticle samples, were employed in four kinds of hydrophilic sedimentary rocks (Boise sandstone, Berea sandstone, Texas Cream limestone, and Ordos sandstone) to investigate the flow enhancement of nanoparticle dispersion in micro-scale porous medium, and the system used in this work is summarized in Figure 1, which shows (a) a sketch of experimental set-up for coreflood in sedimentary rocks, (b) a sketch of no-slip flow and slip flow in a capillary tube, (c) a droplet of decane on a flat Ordos sandstone surface to show the contact angle of 21 degrees, (d) a droplet of decane on a flat Ordos tight sandstone surface to show the contact angle of 23 degrees, and TEM images of (e) 5 nm hydrophilic nanoparticles, (f) 10 nm hydrophobic nanoparticles (HNPs-1), and (g) 15 nm hydrophobic nanoparticles (HNPs-2).

The flow enhancement of nanoparticle dispersion in this work explores the feasible application in oil reservoirs, especially for low permeability reservoirs, since it needs high pressure to inject water into the rocks. Slip flow of nanoparticle dispersion has potential to reduce injected pressure or enhance flow rate, which can improve the performance of water flooding.

## Results

### A model with slip length correction for Darcy flow

During fluid flow in reservoirs, the fluid velocity is typically determined by rock permeability, and Darcy's law is usually employed to describe the fluid flow behavior in reservoir rocks at micro-scale, which is
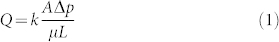
where *Q* is the flow rate, Δ*p* is the pressure difference over a core length of *L*, *k* is core permeability, *A* is cross-sectional area of the core, and *μ* is the fluid viscosity. In micro-scale, the fluid actually flows through a series of pore throats, just like channels or pipes. Based on the rock types, those channels can have diameters between microns to tens of nanometer. For fluid flow calculation through tubes, no-slip boundary is usually assumed and it results in the Hagen-Poiseuille equation, which is
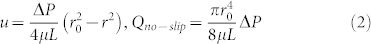
where *r*_0_ is the pipe radius. However, the no-slip boundary assumption is not always true in some circumstances, such as, at very low pressure or when the surface is not perfectly hydrophilic for the flowing phase. A more common approximation for slip fluid with Navier boundary condition^35^ is
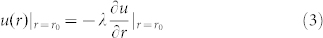
where *λ* is called the slip length. With equation (3), the flow velocity in the tube can be calculated by equation (4):
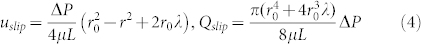
Therefore, the flow enhancement, *E*, which is the ratio of the flow rate with slip, *Q_slip_*, to the flow rate with no-slip, *Q_no-slip_*^36^, in a capillary tube is determined by the slip length, as described in equation (5):
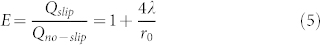
The flow enhancement described in equation (5) is limited to constant pressure difference between the inlet and outlet of capillary tube, so the generalized flow enhancement of fluid flow in porous medium can be expressed by equation (6):


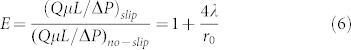


### Flow enhancement of hydrophilic silica nanoparticles

**Figure 2:**
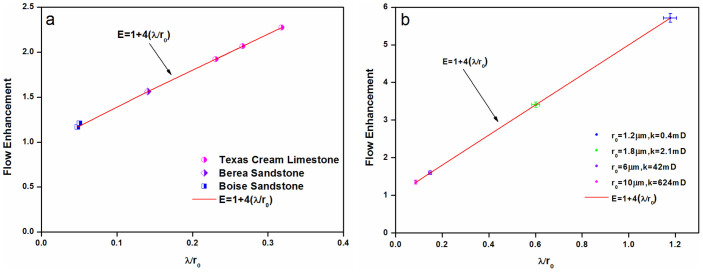
Flow enhancement of water-based nanoparticle dispersions in microscale sedimentary rocks. (a) 5 nm hydrophilic nanoparticle dispersions in Texas Cream limestone, Berea sandstone and Boise sandstone. (b) 10 nm hydrophobic HNPs-1 dispersions in Ordos sandstone and tight sandstone.

The coreflood experiments of 5 nm 3M® silica nanoparticles with concentrations of 18.64 and 5 wt% in different reservoir cores (Texas Cream limestone, Layer-Berea sandstone, and Boise sandstone) were summarized in Table 1. All injected fluids were thermostated to maintain constant temperature of 20 ± 0.2°C. The bulk viscosities at 20°C are 1, 2.5, 1.25 mPa·s for brine, 18.64 wt% nanoparticle dispersion, and 5 wt% nanoparticles dispersion, respectively. The porosities of cores, *ϕ*, as determined by the ratio of pore volume and core bulk volume, are from 0.220 to 0.290, and the permeability is ranging from 10 to 921 mD. Since deionized water will result in clay swelling in rocks, all fluids injected into the rocks were with 3 wt% NaCl, including nanoparticle dispersion and post-flush brine, to avoid clay swelling and formation damage. For each experiment, the flow enhancement and slip length of nanoparticle dispersion were calculated by equation (6), shown in Figure 2a. We observed that the ratio of slip length to pore-throat radius is larger for the rocks with lower permeability, that is, λ/*r*_0_ in Texas Cream limestone (0.231 ~ 0.318) is larger than that in Berea sandstone (0.141), meanwhile, Boise sandstone has the smallest value of λ/*r*_0_ (0.048 ~ 0.050). The sedimentary rocks, unlike capillary tubes or channels with uniform radius, have wide ranges of pore-throat radius and grain sizes, so the values of *r*_0_ used in this work are average pore-throat radius.

### Flow enhancement of hydrophobic silica nanoparticles (HNPs-1)

In order to better understand the slip flow of nanoparticle dispersion in reservoir rocks, coreflood experiments by employing another two kinds of hydrophobic nanoparticle samples (HNPs-1 and HNPs-2) under different flow rates were further conducted in Ordos sandstones. The temperature of target reservoirs in Ordos Basin is 50°C, thus all injected fluids were thermostated to maintain constant temperature of 50 ± 0.05°C. Table 2 lists the value of porosity, which increase from *ϕ* = 0.081, for Ordos tight sandstone (with permeability 0.4 mD), to *ϕ* = 0.275 for Ordos sandstone (with permeability 625 mD), indicating there is strong correlation between porosity and permeability for Ordos sandstones. The volume flow rates, *Q*, for two fluids, brine (deionized water with 1 wt% NaCl) and nanoparticle dispersion, are compared. The bulk viscosities at 50°C are 0.5, 0.51, 0.61 mPa·s for brine, 0.2 wt% HNPs-1, and 0.2 wt% HNPs-2, respectively. The small viscosity difference between brine and nanoparticle dispersions is due to the low concentration of nanoparticle dispersions.

**Figure 3:**
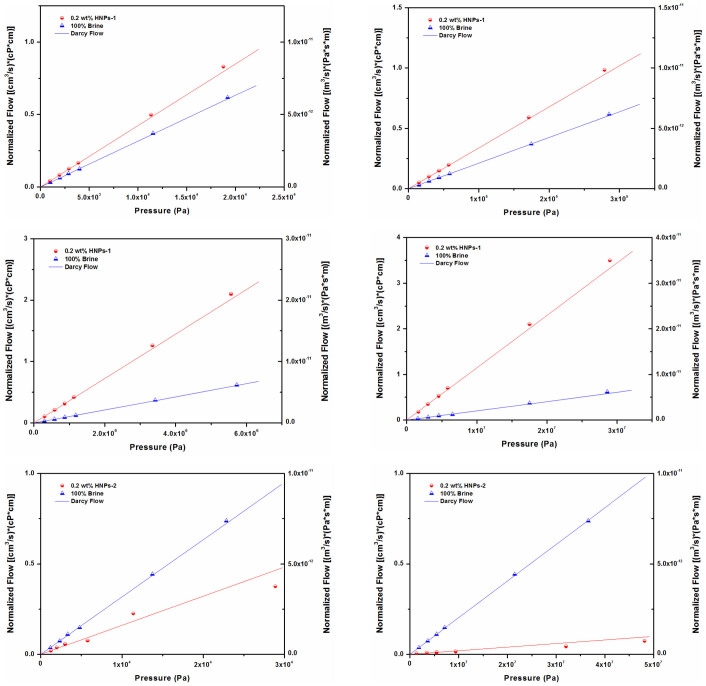
Normalized flow rate *vs* pressure of hydrophobic HNPs-1 & HNPs-2 in Ordos sandstone and tight sandstone, where flow rate is normalized for bulk viscosity and core length. (a) HNPs-1 in core with 10 *μ*m pore-throat radius and 624 mD permeability, (b) HNPs-1 in core with 6 *μ*m pore-throat radius and 42 mD permeability, (c) HNPs-1 in core with 1.8 *μ*m pore-throat radius and 2.1 mD permeability, (d) HNPs-1 in core with 1.2 *μ*m pore-throat radius and 0.4 mD permeability, (e) HNPs-2 in core with 10 *μ*m pore-throat radius and 625 mD permeability, (f) HNPs-2 in core with 1.2 *μ*m pore-throat radius and 0.4 mD permeability. Blue lines are calculated for Darcy flow with no slip for each case where the porosity and permeability were determined independently. Red lines are from least-squares fitting of the data. Flow rate are shown in Darcy units on the left axis and in MKS units on the right axis.

Figure 3 compares the flow of water-based hydrophobic nanoparticle dispersion and brine in the same core with different pressure gradients. The flow rate is normalized by the core length and the bulk viscosity of each phase, which allows brine and nanoparticle dispersion to be compared. Figure 3a presents the data for the core with the largest pore-throat radius, 10 *μ*m, and the highest permeability, 624 mD, showing that the slopes for HNPs-1 and brine are distinguishable. The flow enhancement is 1.36 ± 0.059, where the error is the standard deviation calculated from the propagated errors of the slopes from the linear degradations of the flow rate data for the two liquids. The blue line is the calculated line from Darcy's Law using equation (1), and it virtually overlaps the brine data, indicating that brine has negligible slip flow. Figure 3b shows the normalized flow rate data for the core with relatively larger pore-throat radius, which is 6 *μ*m with permeability of 42 mD. A significant amount of slip flow is evident, and the ratio of slopes for HNPs-1 and brine shows that the flow enhancement is 1.6 ± 0.102, which is higher than that in core with pore-throat radius of 10 *μ*m. Figure 3c presents the normalized flow rate data for the core with relatively smaller pore-throat radius, which is 1.8 *μ*m with permeability of 2.1 mD. A higher amount of flow enhancement is obtained, with HNPs-1 has more than three times normalized flow rate of brine. Figure 3d shows the normalized flow rate data for the core with the smallest pore-throat, which is 1.2 *μ*m in radius, with a lowest permeability of 0.4 mD. The slopes are now drastically different for HNPs-1 and brine, showing the flow enhancement is 5.74 ± 0.554. Figure 3 demonstrates that the flow enhancement increases with the decrease of pore-throat radius for nanoparticle dispersions transport in micro-scale hydrophilic sedimentary rocks, meanwhile, the brine data follow Darcy's Law within experimental error, which indicates that brine has negligible slip flow even for the smallest pore-throat radius of 1.2 *μ*m. One would expect no-slip flow for brine, and the agreement between the brine and the slopes for the Darcy's Law is the guarantee on the experimental accuracy. The ratio of slip length to pore-throat radius for coreflood, determined by equation (6), is shown in Figure 2b. We also observed that the ratio of slip length to pore-throat radius is larger for rocks with smaller pore-throat radius, that is, λ/*r*_0_ in Ordos tight sandstone with 1.2 *μ*m pore-throat radius (1.178 ± 0.028) is larger than that in core with 1.8 *μ*m pore-throat radius (0.602 ± 0.017), meanwhile, Ordos sandstone has even smaller values of λ/*r*_0_, those are 0.148 ± 0.007, 0.086 ± 0.002 in cores with pore-throat radius of 6 and 10 *μ*m, respectively.

## Discussion

**Figure 4:**
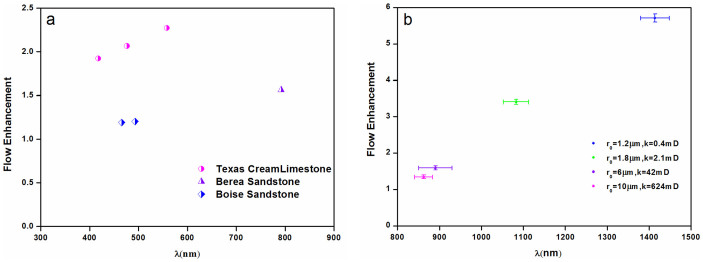
Flow enhancement *vs* slip length of nanoparticle dispersion. (a) 5 nm hydrophilic nanoparticle dispersions in Texas Cream limestone, Berea sandstone and Boise sandstone. (b) 10 nm hydrophobic HNPs-1 dispersions in Ordos sandstone and tight sandstone.

The equation (6) indicates that, if there is a characteristic slip length for nanoparticles with a given size and surface coating, the flow enhancement would be larger when nanoparticles transport in sedimentary rocks with smaller pore-throat radius. Seen from Figure 4a, although the slip lengths of 5 nm hydrophilic nanoparticles are not exactly the same, they are of order 500 nm (462 nm ~ 636 nm), except for the Layered-Berea sandstone (843 nm) caused by tight cluster. The slip lengths of 10 nm hydrophobic nanoparticles, shown in Figure 4b, are of order 1000 nm (862 nm ~ 1414 nm). It suggests that a characteristic slip length exists for these nanoparticles, independent of the lithology or nanoparticle concentration. The deviation of slip length is due to nonuniformity of pore-throat radius of sedimentary rocks. Meanwhile, the flow enhancement increases with decreasing pore-throat radius of sedimentary rocks, which further proves the hypothesis of equation (6). Natural porous media and nanoparticles have surface charge and accompanied zeta potential, which has a huge implication in terms of wettability, and the EDL free energy affects wettability by triggering a hydrophilicity-inducing tendency, with the effect showing a larger magnitude for larger ionic concentrations^39^. However, the wettability change of rocks by 3M® nanoparticles flooding can be negligible, while the wettability of Ordos sandstone is changed from hydrophilic surface to a less hydrophilic state by HNPs-1 nanoparticles flooding, which is due to the adsorption of nanoparticles on the rock surface. The flow enhancement of nanoparticle dispersion in microscale sedimentary rocks may be caused by nanoparticle-rock interactions that enhance particle layering^40^,^41^,^42^ and this structure in an inhomogeneous manner can significantly improve confined fluid mobility, corresponding to states with higher excess entropy but lower entropy than those with the natural structure profile. For one thing, the shear stress somehow enables particles to arrange themselves in a more ordered fashion; for another, the nanoparticles absorbed at the rock surfaces features as lubricant, resulting in slip flow and higher mobility.

### Independence of slip length on shear rate

**Figure 5:**
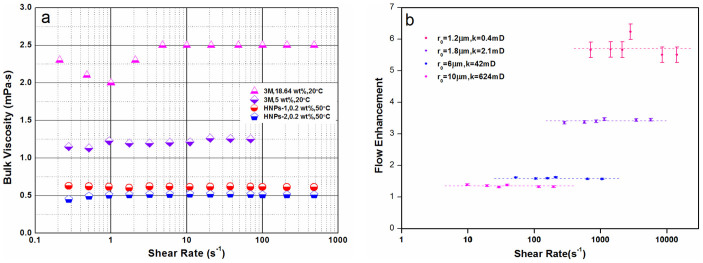
Dependence of slip length of nanoparticle dispersions on shear rate. (a) Dependence of bulk viscosity on shear rate for aqueous dispersions of 5 nm hydrophilic nanoparticles (20°C), 10 nm HNPs-1 (50°C) and 15 nm HNPs-2 (50°C). (b) Flow enhancement *vs* shear rate for HNPs-1 in Ordos sandstone and tight sandstone, indicating that flow enhancement is independent of shear rate under experimental conditions of this work. The shear rate is changed by increasing the flow rate from 0.5 to 10 mL/min.

Many researchers investigated the dependence of slip length on shear rate, however, the results were in remarkable disagreement. Slip length was reported to increase with shear rate for water in a hydrophobic microchannel^23^, and theory also indicates that Newtonian fluids will exhibit non-Newtonian behavior under sufficiently high shear rate^37^; but slip flow of water through silica colloidal crystals was independent of shear rate over a range of fluid velocities from 0.7 to 5.8 mm/s^15^. Prior to coreflood experiment for investigation the dependence of slip length on shear rate, the effect of shear rate on bulk viscosity of nanoparticle dispersions is determined, as shown in Figure 5a. The bulk viscosities show little or no shear-rate dependence in the range between 0.1 and 1000 s^−1^, that is, the nanoparticle dispersion reveals rheology as Newtonian fluids, which indicates the rheological behavior of nanoparticle dispersion is unlike that of typical concentrated colloidal dispersions. In this work, the dependence of slip length on shear rate was investigated in four Ordos sandstones by increasing the flow rate of HNPs-1 from 0.5 to 10 mL/min, which gives a 20-fold variation in fluid velocity. The results are shown in Figure 5b, indicating no detectable shear rate dependence under experimental conditions of this study.

### Flow degradation of HNPs-2

Both 3M® hydrophilic nanoparticle and HNPs-1 hydrophobic nanoparticle exhibit slip flow behavior during transport through sedimentary rocks, however, the phenomenon of flow degradation was observed for HNPs-2 nanoparticle in Ordos sandstones. The flow enhancements, (*m*(NP)/*m*(water), the same concept used for analysis as 3M® nanoparticle and HNPs-1, were 0.54 and 0.10 in Ordos sandstones with pore-throat radius of 10 *μ*m and 1.2 *μ*m, respectively, shown in Figure 3e & f. The main reason is the high retention/adsorption of HNPs-2 nanoparticles at the rock grain surfaces, which reduces the pore-throat radius and results in bridge block and formation damage, indicating not all nanoparticle dispersions result in flow enhancement at microscale. The phenomenon of flow degradation was also observed in our former research^29^, i.e., commercial Nanorods, in-house synthesized nanoclusters with PVP (polyvinylpyrrolidone) or TMAOH (tetramethylammonium hydroxide) as surface coating. The flow physics involved in nanoparticle flow in sedimentary rocks is quite complex. Whether one can generalize the DLVO (Derjaguin-Landau-Verwey-Overbeek) theory to gain insights into such phenomena^43^,^44^,^45^,^46^ (e.g., van der Waals force, electrostatic repulsion, acid-base interaction and Born repulsion), however, nanoparticle retention in sedimentary rocks remains an open question (e.g., steric repulsion, nanoparticle transport in reservoir rocks with extremely high ionic concentration, 8 wt% NaCl + 2 wt% CaCl_2_)^47^.

## Methods

### Nanoparticle materials

Hydrophilic silica nanoparticles with nominal diameter of 5 nm were obtained from 3M® (St. Paul, MN, USA) as 18.64 wt% aqueous dispersion, and were diluted to the desired concentration. The coatings consist of polyethylene glycol (with about 7 EG units), which is covalently attached to the silica surface through silicon-oxygen-silicon bonds. The coating allows the individual nanoparticles stay dispersed in water/brine without aggregation, even under high ion strength conditions (10 wt%, or 1710 mM NaCl). Two kinds of hydrophobic silica nanoparticles with proprietary surface coating were supplied by Tsinghua University (Beijing, P.R.China) as 0.2 wt% aqueous dispersion. The surface coatings of nanoparticles and extra 0.1 wt% carboxylate surfactant in aqueous phase enable hydrophobic nanoparticles dispersed in water. The 3M® nanoparticle dispersions can be stale for months, while the hydrophobic nanoparticles can be stable in aqueous dispersion for about 48 hours.

### Microscale sedimentary rocks

Boise sandstone, Berea sandstone, and Texas Cream limestone are common sedimentary rocks in USA, and they were widely used in coreflood experiments. Boise sandstone, obtained from a quarry in Idaho, has large grain size and coarse texture. Its grains are more loosely packed than Berea sandstone and flake away easily. Berea sandstone, obtained from a quarry in Ohio, has fairly uniform grain size and smooth texture. The ‘Layer-Berea sandstone’ has some thin shale layers, which decreased the core porosity and permeability. The Texas Cream Limestone was from Texas, and has uniform grain size and smooth texture. Ordos sandstones, with a wide range of permeability from 0.4 mD to 625 mD, were oilfield reservoir rocks and supplied by PetroChina. The coring is shown in Supporting Information. Core plugs 2.54 cm in diameter and 7.62 cm in length were used for all of the coreflood experiments.

### Bulk viscosity measurement

The bulk viscosity of 3M® hydrophilic nanoparticle dispersion was measured by using Advanced Rheometric Expansion System LS-1 rheometer (TA Instruments, DE, USA). The RS6000 rotary rheometer (HAAKE, Germany) was employed to measure the bulk viscosity of hydrophobic nanoparticle dispersions.

### Nanoparticle morphology

Transmission electron microscopy (TEM) was used to observe the morphology of the nanoparticles. The experiments were performed on a FEI TECNAI G2 F20 X-TWIN TEM using a high-angle annular dark field detector. The samples were prepared using a “flash-freezing” technique, in which a 200 mesh carbon-coated copper TEM grids were cooled using liquid nitrogen and then dipped into dilute aqueous nanoparticle dispersion^38^. The sample was immediately dried using a Virtis Advantage Tray Lyophilizer (Virtis Company, NY, USA) with 2 hours of primary drying at −40°C followed by a 12 hour ramp to +25°C and then 2 hours of secondary drying at 25°C. In this manner, the aggregation of the nanoparticles, caused by capillary forces during drying of the liquid on the TEM grid could be avoided.

### Flow rate control

Flow rates of 3M® nanoparticle dispersions were generated by a Isco 1000D syringe pump (Teledyne Isco, Lincoln, NE, USA), and a Isco 500D syringe pump (Teledyne Isco, Lincoln, NE, USA) was employed to control the flow rates of HNPs-1 & HNPs-2 nanoparticle dispersions. The pressure differences between inlet and outlet of cores were measured by high-accuracy transducers PX409-001GI, PX409-100GI, and PX409-5.0KGI (Omega, Stamford, CT, USA), and the effluent was collected in ∅10 × 75 mm borosilicate glass disposable culture tubes (Thermo-Fisher Scientific, Waltham, MA, USA).

### Contact angle measurement

The contact angle goniometer JPSY-360 (Beijing United Test Co., Beijing, China) was used to monitor and measure the contact angle of decane droplet on Ordos sandstone surfaces immersed in deionized water with 3 wt% NaCl. Curve fitting was carried with Origin software (OriginLab, Northhampton, MA, USA), and Microsoft Visio (Microsoft, Seattle, WA, USA) was employed to draw figures.

## Author Contributions

H.Y., S.C., S.L.B. and C.H. designed the experiments and supervised the project; H.Y. and Y.H. wrote the manuscript; H.Y., Y.H., P.L. and T.Z. conducted coreflood experiments; S.L. measured bulk viscosity; C.W. performed rock characterization; C.W.B. synthesized surface coating materials; E.R. and S.D. analyzed data.

## Supplementary Material

Supplementary Information Supplementary Information

## Figures and Tables

**Figure 1:**
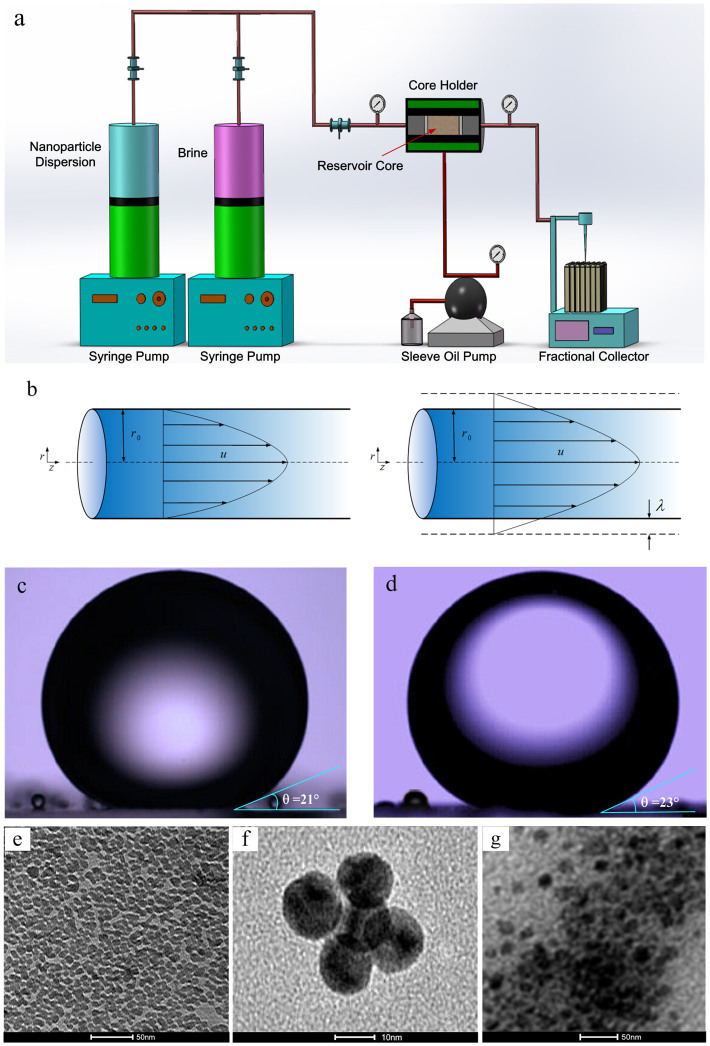
Summary of the type of system studied in this work. (a) A sketch of experimental set-up for coreflood in sedimentary rocks. (b) A sketch of no-slip flow and slip flow in a capillary tube. (c) A droplet of decane on a flat Ordos sandstone surface to show the contact angle of 21 degrees. (d) A droplet of decane on a flat Ordos tight sandstone surface to show the contact angle of 23 degrees, and TEM images of (e) 5 nm hydrophilic nanoparticles, (f) 10 nm hydrophobic nanoparticles (HNPs-1), and (g) 15 nm hydrophobic nanoparticles (HNPs-2).

**Table 1:** Summary of Coreflood Experimental Conditions under 20°C and Measured Parameters of 3M® Nanopartcle Dispersion in Sedimentary Rocks: Pore-Throat Radius (*r*_0_)^47^, Permeability (*k*), Porosity (*ϕ*), Nanoparticle Concentration (*C*_0_), Flow Rate (*Q*), Pressure Difference for Nanoparticle Flow (Δ*P(NP*)), Pressure Difference for Brine Flow (Δ*P*(*Brine*)), Bulk Viscosity (*μ*), Shear Rate (*γ*), Flow Enhancement (E), Slip Length (*λ*), Ratio of Slip Length to Pore-Throat Radius (*λ*/*r*_0_)

Porous Medium	*r*_0_ (μm)	*k* (mD)	*ϕ*	C_0_ (wt%)	*Q* (m/min)	ΔP(NP) (×10^3^ Pa)	ΔP(Brine) (×10^3^ Pa)	μ_bulk_ (cP)	γ (s^−1^)	E	λ (nm)	λ/r_0_
Texas Cream Limestone 1	2	15	0.29	18.64	2	796	413.9	2.5	244	1.923	462	0.231
Texas Cream Limestone 2	2	10	0.22	18.64	2	1190	577.9	2.5	344	2.066	533	0.267
Texas Cream Limestone 3	2	10	0.22	18.64	2	1190	525.4	2.5	344	2.273	636	0.318
Berea Sandstone	6	136	0.22	18.64	2	87.8	56.19	2.5	93	1.563	843	0.141
Boise Sandstone 1	10	921	0.28	18.64	1	6.48	5.445	2.5	16	1.191	476	0.048
Boise Sandstone 2	10	867	0.29	5	1.1	3.79	3.151	1.25	18	1.202	505	0.050

**Table 2:** Summary of Coreflood Experimental Conditions under 50°C and Measured Parameters of HNPs-1 & HNPs-2 in Ordos Sandstones: Pore-Throat Radius (*r*_0_)^a^, Permeability (*k*), Porosity (*ϕ*), the Calculated Slope for No-Slip Darcy Flow (*m*(DL)), Measured Slopes for Flow Rate *vs* Pressure for Water and Nanoparticle (*m*(water) and *m*(NP))^b^, Shear Rate (γ)^c^, Flow Enhancement (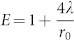), and Slip Length (*λ*)

Porous Medium	*r*_0_ (μm)	k (mD)	ϕ	m(DL) (×10^−8^)	m(water) (×10^−8^)	m(NP) (×10^−8^)	γ (s^−1^)	E	λ (nm)	λ/r_0_
Ordos Sandstone 1^d^	10	624	0.274	3162	3150 ± 77	4250 ± 27	10–195	1.345 ± 0.009	862 ± 21	0.086 ± 0.002
Ordos Sandstone 2^d^	6	42	0.14	212.8	212.4 ± 5.28	33.9 ± 5.64	53–1051	1.593 ± 0.026	890 ± 40	0.148 ± 0.007
Ordos Tight Sandstone 1^d^	1.8	2.1	0.095	10.64	10.61 ± 0.12	36.24 ± 0.71	285–5704	3.406 ± 0.067	1083 ± 30	0.602 ± 0.017
Ordos Tight Sandstone 2^d^	1.2	0.4	0.081	2.03	2.03 ± 0.09	11.58 ± 0.23	708–14155	5.713 ± 0.114	1414 ± 34	1.178 ± 0.028
Ordos Sandstone 3^e^	10	625	0.275	3167	3190 ± 86	1710 ± 312	10–194	0.539 ± 0.099		
Ordos Tight Sandstone 3^e^	1.2	0.4	0.14	2.03	2 ± 0.006	0.2 ± 0.004	538–10766	0.101 ± 0.019		

^a^Pore-throat radius of Ordos core is average pore-throat radius, taken from mercury porosimetry on a representative sample from each block. The distribution of pore-throat radius is shown in Fig. S3 of Supporting Information.

^b^The slopes are in units of cm^4^·cP/(Pa·s), where cP is the viscosity in centipoise and 1 cP = 1 mPa·s.

^c^The shear rate is changed by changing the flow rate from 0.5 to 10 mL/min for each core.

^d^The fluid injected into the core is HNPs-1.

^e^The fluid injected into the core is HNPs-2.
