# Supplementary material for: Flow enhancement of water-based nanoparticle dispersion through microscale sedimentary rocks
Source: Sci Rep. 2015 Mar 3;5:8702. doi: 10.1038/srep08702 (PMC4346797; doi:10.1038/srep08702)
Supplement: Supplementary Information [file srep08702-s1.pdf]

Supplementary Information

Title: Flow enhancement of water-based nanoparticle dispersion through microscale sedimentary rocks

Haiyang Yu[1,*], Youwei He[1,*], Peng Li[1], Shuang Li[1], Tiantian Zhang[2], Elena Rodriguez-Pin[2], Song Du[3], Chenglong Wang[4], Shiqing Cheng[1], Christopher W. Bielawski[5], Steven L. Bryant[2], and Chun Huh[2]

[1]MOE Key Laboratory of Petroleum Engineering, China University of Petroleum Beijing, Beijing, 102249, P.R. China.

[2]Department of Petroleum and Geosystems Engineering, University of Texas at Austin, Austin, TX, 78712, USA.

[3]Department of Petroleum Engineering, Texas A & M University, Collage Station, TX, 77843, USA.

[4]Yanchang Oilfield Co., Ltd. Yan'an, Shanxi, 716000, P.R. China.

[5]Department of Chemistry and Biochemistry, University of Texas at Austin, Austin, TX, 78712, USA.

[*]These authors contributed equally to this work. Correspondence and requests for materials should be addressed to H.Y (email: haiyangyu@utexas.edu)

1. Coring

Coring of Ordos sandstones was shown in **Figure S1**. Four reservoir rocks were directly cored during drilling in four wells with different depths.

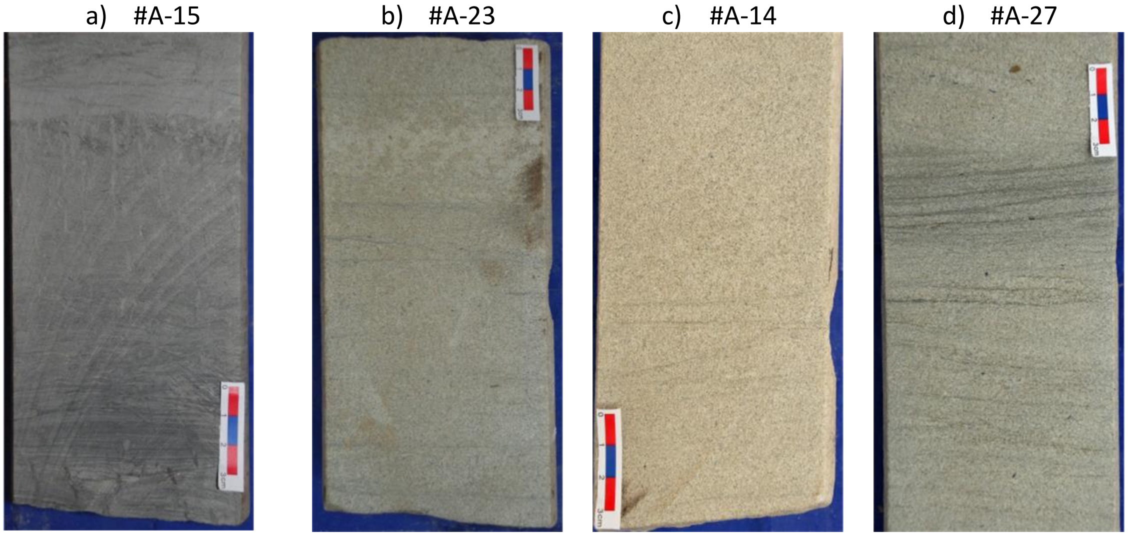

Figure S1. **Coring of Erdos sandstones from four wells with different TVD (true vertical depth), oilfield reservoir rocks.** (**a**) Tight sandstone with k= 0.4 mD and TVD 3531 m from well A-15. (**b**) Tight sandstone with k=2.1 mD and TVD 3541 m from well A-23. (**c**) Sandstone with k=41.6 mD and TVD 3524 m from well A-14. (**d**) Sandstone with k=624 mD and TVD 2526 m from well A-27.

2. Coreflood Experiments Procedures

2.1 Boise sandstone, Berea sandstone, and Texas Cream limestone

Since there are no hydrocarbons in these rocks, the coreflood experiment procedures are:

(i) The core was dried in the thermostat at 20 ℃ for more than ten days and weighted in air;

(ii) The core was then vacuumed for twenty-four hours and saturated with water (having the same salinity with nanoparticle dispersion, 3 wt% NaCl), and weighed again. Porosity was calculated by the weight difference and the core dimensions;

(iii) The constant-rate injection of water (the same as above) was carried out to obtain the permeability of the core, calculated by Darcy's law;

(iv) Nanoparticle dispersion was injected through the core with the same flow rate of water.

2.2 Ordos sandstone & tight sandstone

Hydrocarbons exist in Ordos sandstones, shown in **Figure S2**, since the cores were directly cored during drilling in the target reservoirs. The coreflood experiment procedures are:

(i) The core was firstly washed by toluene and water (1 wt% NaCl) until no hydrocarbons existence in the core;

(ii) The core with 100% water saturation was then dried in the thermostat at 50 ℃ for more than ten days and weighted in air;

(iii) The core was then vacuumed for twenty-four hours and saturated with water (having the same salinity with nanoparticle dispersion, 1 wt% NaCl), and weighed again. Porosity was calculated by the weight difference and the core dimensions;

(iv) Different flow rates of water (0.5, 1, 1.5, 2, 6, 10 mL/min) were injected into the core, and the corresponding steady-state pressure differences were recorded;

(v) Nanoparticle dispersion was injected through the core with the same flow rate of water (0.5, 1, 1.5, 2, 6, 10 mL/min).

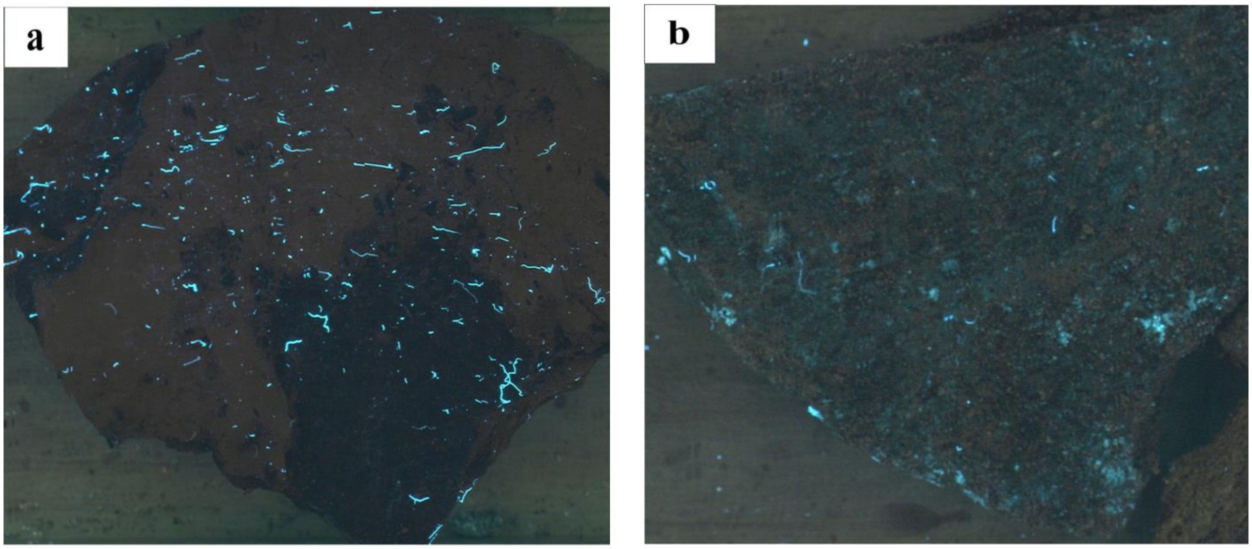

Figure S2. **Hydrocarbons identification of reservoir rocks by Scanning Fluorescence Technique, and the fluorescence represents hydrocarbons.** (**a**) Tight sandstone from well A-15. (**b**) Sandstone from well A-14.

3. Pore-Throat Radius

The pore-throat radius used in this work is average value for each core. The distribution of pore-throat radius of Ordos sandstone is measured by mercury porosimetry ASPE-730 Automated System for Pore Examination (Coretest Systems, Morgan Hill, CA, USA), shown in **Figure S3**.

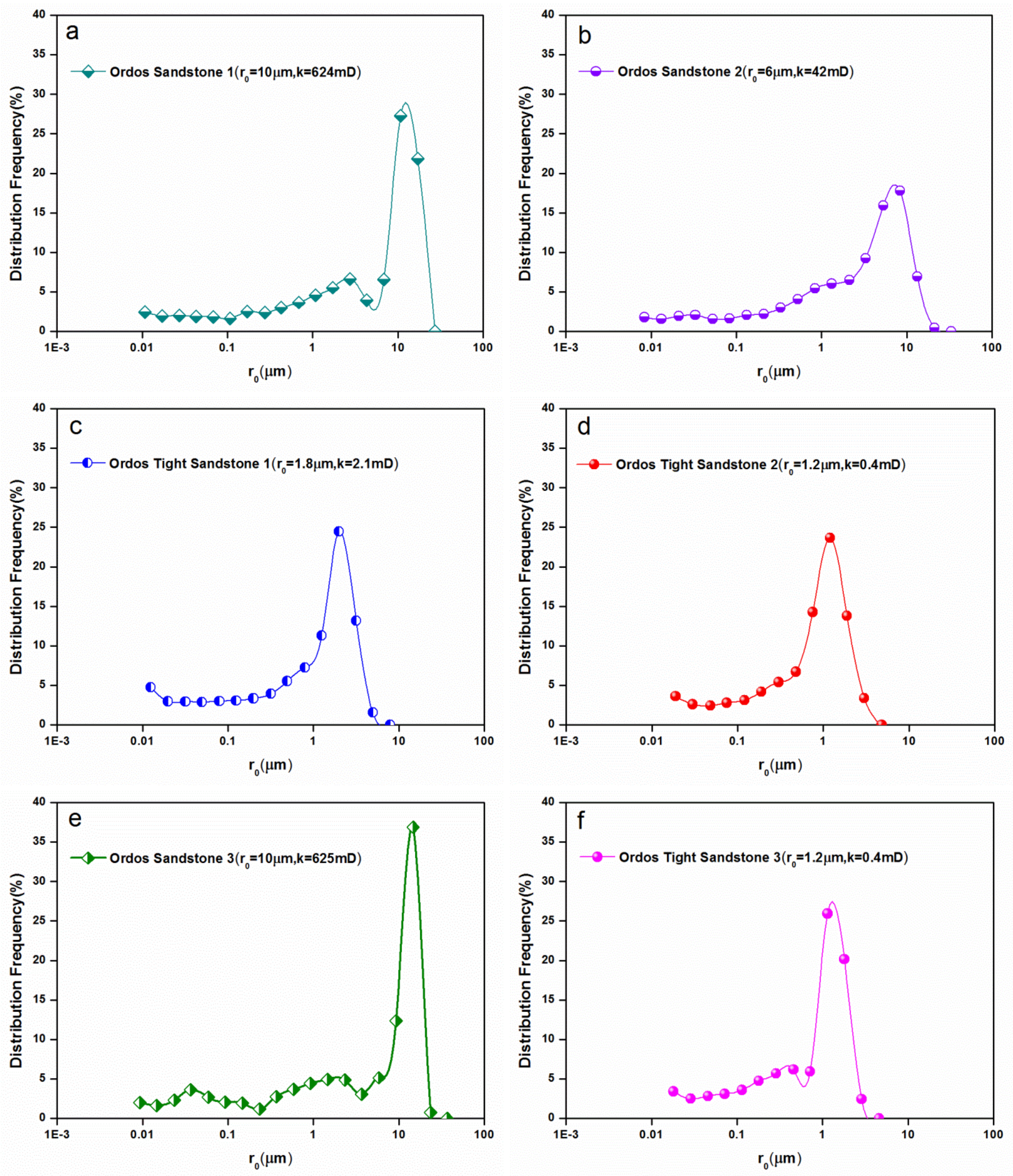

Figure S3. Pore-throat radius distribution of Ordos sandstone & tight sandstone by mercury porosimetry. (**a**) Ordos sandstone with permeability 624 mD, (**b**) Ordos sandstone with permeability 42 mD, (**c**) Ordos tight sandstone with permeability 2.1 mD, (**d**) Ordos tight sandstone with permeability 0.4 mD, (**e**) Ordos sandstone with permeability 625 mD, (**f**) Ordos tight sandstone with permeability 0.4 mD.

4. Dependence of bulk viscosity on nanoparticle concentration (5 nm 3M® silica)

Figure S4 shows the bulk viscosities of nanoparticle dispersions measured with different nanoparticle concentrations. The bulk viscosities of all the nanoparticle dispersions are less than 3 mPa·s, even at large particle loadings, i.e. 18.64 wt%. The bulk viscosities at 20 ℃ are 1, 2.5, 1.25 mPa·s for brine, 18.64 wt% nanoparticle dispersion, and 5 wt% nanoparticles dispersion, respectively.

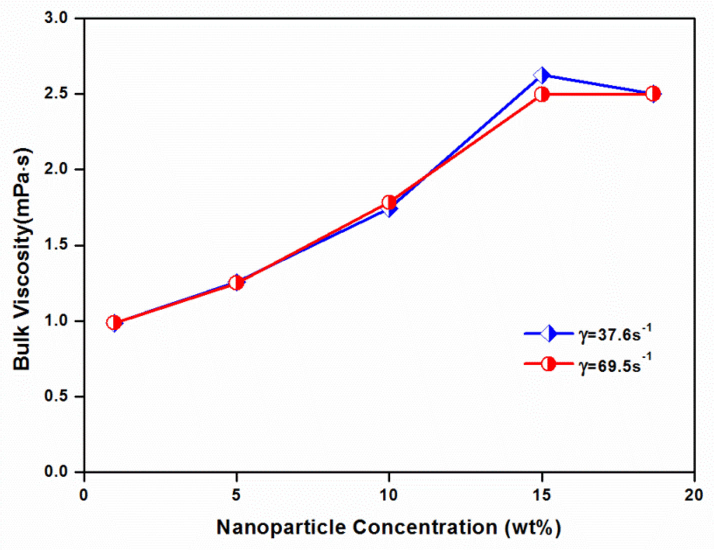

Figure S4. **Dependence of bulk viscosity on nanoparticle dispersions (5 nm 3M® silica) under 20 ℃.**
